# Neutrophil Extracellular Traps in Pediatric Infections: A Systematic Review

**DOI:** 10.3390/cimb47120999

**Published:** 2025-11-28

**Authors:** Anastasia Stoimeni, Nikolaos Gkiourtzis, Vera Karatisidou, Nikolaos Charitakis, Kali Makedou, Despoina Tramma, Paraskevi Panagopoulou

**Affiliations:** 1 4th Department of Pediatrics, School of Medicine, Faculty of Health Sciences, Aristotle University of Thessaloniki, Papageorgiou General Hospital, 56403 Thessaloniki, Greece; gkiourtzisnikolaos@gmail.com (N.G.); vkaratisidou@gmail.com (V.K.); dtramma@auth.gr (D.T.); ppanagopoulou@auth.gr (P.P.); 2 Saint Luke’s Private Clinic, 55236 Thessaloniki, Greece; nickcharitakis1996@gmail.com; 3 Laboratory of Biochemistry, AHEPA University Hospital, Department of Health Sciences, School of Medicine, Aristotle University of Thessaloniki, 54124 Thessaloniki, Greece; kalimakedou@gmail.com

**Keywords:** NETs, NETosis, pediatrics, neutrophils, infection, biomarkers, systematic review

## Abstract

Background: Neutrophil extracellular traps (NETs) are granule- and nucleus-derived structures that support innate immunity. While the contribution of NETs to adult infections and autoimmune diseases is well studied, evidence in children is still inconsistent. This review aimed to summarize current findings on NETs in pediatric infections. Methods: This study followed the Cochrane Handbook for Systematic Reviews of Interventions and adhered to the PRISMA guidelines. A search was conducted in major databases (MEDLINE/PubMed and Scopus) from inception until 5 September 2025. The study quality was evaluated using the modified Newcastle–Ottawa Scale. Results: Eleven studies were included in the systematic review. In respiratory disease, the role of NETs was well described and their formation correlated with severity. Patients with febrile urinary tract infections showed elevated urinary NET-associated markers. In COVID-19 infection, NET levels were unchanged in uncomplicated cases but elevated in multisystem inflammatory syndrome in children. Findings in sepsis were inconsistent. Conclusions: This systematic review presents the published evidence on NET formation in the pediatric population, assessing the current knowledge and identifying the gaps to guide research. Future studies should aim to standardize NET detection methods, evaluate their prognostic value in large prospective cohorts, and explore the various NET-associated mechanisms in children.

## 1. Introduction

Neutrophil extracellular traps (NETs) are granule and nuclear structures that have a crucial role in the process of bacterial extracellular death, functioning as an innate immune defense mechanism [1,2,3,4]. However, excessive or dysregulated NET formation has been shown to contribute to tissue inflammation and injury, as well as autoimmunity [5,6,7]. Neutrophil extracellular traps (NET) formation is facilitated by peptidylarginine deiminase 4 (PAD4), a critical enzyme which catalyzes histone citrullination, promotes chromatin decompensation and enables their release [8]. Other pathways contributing to NET formation include reactive oxygen species generation, neutrophil elastase and myeloperoxidase activity, underscoring the complex regulation of their release [3].

The role of NETs in infections and autoimmune conditions in adults has been extensively studied [9,10]. In contrast, evidence in pediatric populations remains limited and heterogeneous. Neonates and children exhibit distinct neutrophil phenotypes compared to adults, including reduced phagocytic capacity and impaired NET formation [11,12]. These differences may impact NET function and lead to different responses to infections, potentially leading to age-specific patterns of host defense, disease susceptibility, and outcomes following infection [13].

The involvement of NETs in pediatric infections has previously been investigated. They have been described in respiratory tract infections and diseases, such as recurrent otitis media and cystic fibrosis and in systemic infections, including neonatal and pediatric sepsis [14,15,16]. While in some settings, NETs appear to have a protective role by limiting microbial growth, in others, they contribute to persistent inflammation and tissue damage, highlighting their dual role in pediatric host defense and disease.

As a result, pediatric NET research is limited by inconsistent reporting of clinical outcomes. In addition, the clinical relevance of NETs in children in not clearly understood, as the correlation between NET formation and disease severity, prognosis, or therapeutic interventions has not been sufficiently investigated. To address this critical gap, the present study aims to provide the first systematic review of pediatric NET studies, to synthesize the reported associations with clinical outcomes, identify knowledge gaps, and help guide the development of age-appropriate interventions targeting NET-mediated inflammation.

## 2. Materials and Methods

### 2.1. Study Registration and Search Strategy

This systematic review followed the Cochrane Handbook for Systematic Reviews of Interventions and adhered to PRISMA guidelines [17]. The protocol was registered in OSF (https://osf.io/c3reb/overview, accessed on 24 November 2025). We searched MEDLINE/PubMed and Scopus databases from their inception until 5 September 2025. A basic search strategy was developed for MEDLINE/PubMed, including all fields of published studies without exception, and modified accordingly for other research engines using the terms: “neutrophil extracellular traps” OR “NETs” AND “child*” OR “adolescent*” OR “infant” OR “neonate” AND “infection” OR “infectious disease”. Reference lists of detected studies were also screened for further eligible studies. PROSPERO and OSF databases were also screened to avoid duplicate reviews. Only English studies were included, with no year restrictions.

### 2.2. Eligibility Criteria

All case–control and/or observational studies examining the presence, role, or effects of NETs in pediatric patients with infectious diseases that met the eligibility criteria described in the predetermined protocol were included in the study. For studies that included mixed adult and pediatric populations, only pediatric data were extracted and included in the analyses. Studies reporting in vitro findings were excluded.

### 2.3. Collection and Extraction of Data

Two independent reviewers (A.S. and N.G.) conducted the literature search and data extraction. Records from databases were imported into a reference management tool (rayyan.qcri.org), with duplicates removed [18]. Titles and abstracts were initially screened, followed by full-text assessment of the remaining articles based on eligibility criteria. Any discrepancies were resolved through discussion with a third author (V.K.) until consensus was reached. Reference lists of included studies were manually reviewed for additional studies. ClinicalTrials.gov, PROSPERO, OSF, and “grey literature” were also searched to identify ongoing studies. Two authors (V.K. and A.S.) independently extracted the baseline characteristics of the included studies using a pre-specified form. Missing data were obtained by contacting the corresponding authors.

### 2.4. Quality Assessment of the Included Studies

Two independent reviewers (A.S. and Ν.C.) assessed the quality of included observational studies, resolving disagreements through consensus, using the modified Newcastle–Ottawa Scale (NOS) [19]. Studies were classified as low (0–3 points), medium (4–6 points), or high quality (7–9 points). For cross-sectional studies, we applied an adapted version of the NOS [20,21,22,23], which modifies selection, comparability, and outcome criteria to suit cross-sectional design characteristics. This tool evaluates three key domains for a total possible score of 10 stars, and the included cross-sectional studies were classified as high quality (7–10 stars), moderate quality (5–6 stars), and low quality (≤4 stars), consistent with thresholds applied in previous systematic reviews [20,21].

### 2.5. Outcome Measurements

According to the pre-specified protocol, the main outcomes included assessment of NETs in pediatric infections through laboratory or clinical measures, and the association between NETs and infection severity, complications, and treatment response.

## 3. Results

### 3.1. Search Strategy Results and Study Characteristics

**Figure 1:**
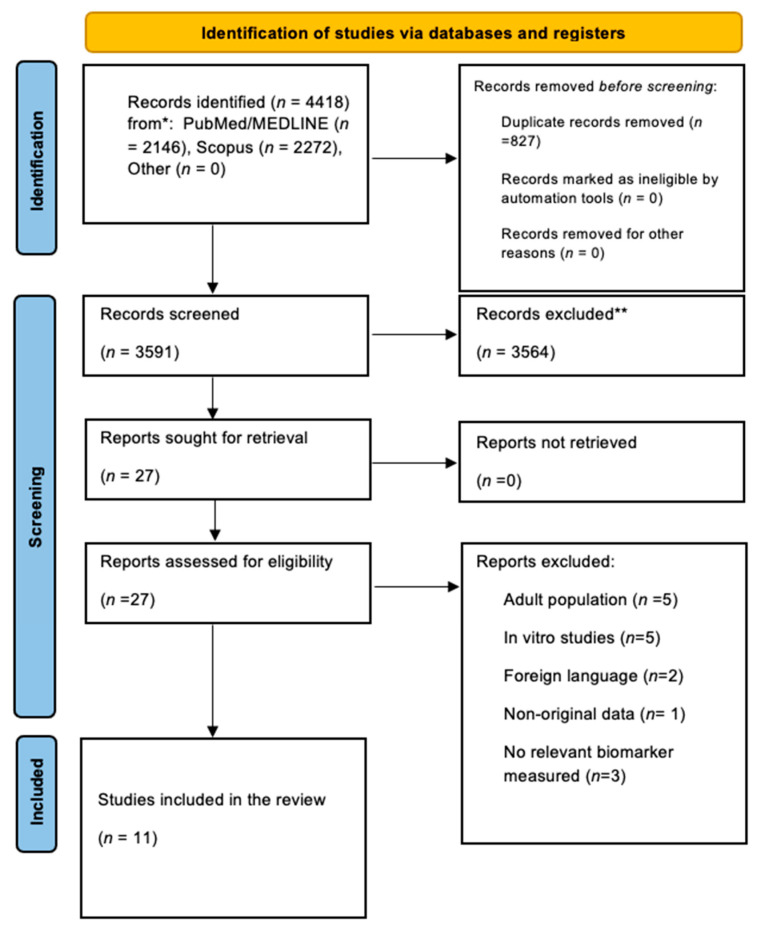
The PRISMA 2020 flow chart.

In total, 4418 records were identified in the initial search. After duplicate removal and title and abstract screening, 27 studies remained for full-text assessment, with 11 studies being eligible for our systematic review (Figure 1, Table A1 and Table A2) [14,15,16,24,25,26,27,28,29,30,31]. The baseline characteristics of the included studies are presented in Table 1.

### 3.2. Quality Assessment of the Included Studies

Six out of 11 studies included in our systematic review were evaluated as “high quality” [15,26,28,29,30,31], while the other five as “medium quality” [14,16,24,25,27]. A summary of the quality assessment is presented in Table 2.

### 3.3. Outcomes

#### 3.3.1. Respiratory Infections

Respiratory infections such as otitis media and chronic airway inflammation were found to be associated with solid evidence for NET involvement. Thornton et al. [27] demonstrated that NETs were present in middle ear effusions in children with recurrent acute otitis media. This finding suggests that NETs may promote the persistence of infection by stabilizing biofilm structures. In a study of cystic fibrosis (CF) patients, King et al. [28] found robust formation of both neutrophil and macrophage extracellular traps in bronchoalveolar lavage (BAL) fluid, with NET expression strongly associated with neutrophil elastase activity. Similarly, Martínez-Alemán et al. [30] reported that Pseudomonas aeruginosa isolates from children with cystic fibrosis induced distinct NET morphologies in vitro. The activation of NETosis in the airway tracts of individuals with CF has been attributed to the worsening lung conditions [32]. In children undergoing bronchoscopy, the presence of NETs was strongly associated with neutrophil elastase activity [28]. Pathogens isolated from children with more severe disease were more likely to promote the development of spread NETs, indicating that modulation of NET architecture by the pathogen may play a role in disease severity.

During the COVID-19 pandemic, NETs were studied in relation to SARS-CoV-2 infection in children. Seery et al. [26] analyzed blood samples from 174 children with COVID-19, 21 with MIS-C, and 40 healthy controls. They found no significant elevation of cfDNA or citrullinated histones compared with controls, although neutrophils displayed altered activation and inhibitory receptor expression. In contrast, Carmona-Rivera et al. [29] showed that NET remnants were markedly elevated in children with MIS-C, while histological examination revealed NET deposition in skin biopsies. These findings suggest that while uncomplicated pediatric COVID-19 is not characterized by NETosis, dysregulated NET responses may contribute to the immunopathology of MIS-C and related syndromes.

#### 3.3.2. Urinary Tract Infections

The role of NETs in pediatric urinary tract infections was addressed in only one study. Krivošíková et al. [31] demonstrated significantly higher urinary levels of extracellular DNA, myeloperoxidase (MPO), and cathelicidin in children with febrile urinary tract infections (UTIs) compared with healthy controls. In their analysis, the authors also reported that elevated extracellular DNA levels positively correlated with fever duration and inflammatory markers, indicating that NET formation may reflect the severity of the infectious process.

#### 3.3.3. Central Nervous System Infections

The effect of NETs has also been investigated in pediatric central nervous system (CNS) infections in one study. Appelgren et al. [24] examined cerebrospinal fluid samples from children and adults with Lyme neuroborreliosis and other CNS infections. The researchers revealed that NETs were more frequently detected in pediatric samples than in adults, and their presence correlated with elevated levels of chemokines and cytokines. Neutrophil extracellular traps detection was also shown to be associated with clinical features such as fever and laboratory findings like pleocytosis, supporting their role in the inflammatory response of CNS.

#### 3.3.4. Sepsis

The involvement of NETosis in sepsis is less consistent. In a cohort of children admitted with meningococcal sepsis, Hoppenbrouwers et al. [15] observed markedly elevated serum MPO-DNA complexes at admission and after 24 h, although these did not correlate with disease severity or outcome. Conversely, two neonatal studies questioned the predictive utility of NET markers. Stiel et al. [14] reported that cord blood levels of cfDNA, neutrophil elastase, and MPO did not differ between neonates who developed early-onset sepsis and matched controls, suggesting limited NET forming capacity at birth. In contrast, Lenz et al. [16] measured plasma markers in preterm infants with suspected sepsis and found that cfDNA and DNase I were significantly elevated in both early- and late-onset sepsis, whereas citrullinated histone H3 and elastase showed no differences between the sepsis group and the control group.

## 4. Discussion

This systematic review demonstrates that NETs have a key role in a wide range of pediatric infections. In pediatric UTIs, elevated urinary levels of extracellular DNA, myeloperoxidase, and cathelicidin have been documented [31]. The absence of key NETosis mediators such as PAD4 or Tamm-Horsfall protein resulted in higher bacterial loads and worse infection outcomes in UTIs [33]. Moreover, NETs may also contribute to chronic inflammation. Findings in CF suggest that while NETs can limit microbial growth, their persistence in tissues may sustain inflammation and damage [30]. The COVID-19 pandemic provided further information regarding NET biology in children. In uncomplicated pediatric COVID-19, no elevation in plasma NET markers was observed [26]. By contrast, in children with MIS-C, NET remnants were markedly elevated, with histological evidence of NET deposition and impaired degradation [29]. These findings suggest that dysregulated NET clearance, rather than overproduction alone, may drive immunopathology in post-infectious inflammatory syndromes, i.e., Kawasaki disease, opening new areas of potential research.

In neonatal fungal infection models, NET formation may provide a crucial line of defense for neonates and young children against specific pathogens [34]. Although the contribution of NETs to severe infections and sepsis remains uncertain, Zhang et al. [35] applied machine learning to pediatric datasets and identified a five-gene NET-related signature that discriminated septic children from controls with high accuracy, while validation in clinical samples confirmed this diagnostic potential.

Compared with adults, where large cohort studies in sepsis and COVID-19 infection have consistently demonstrated marked systemic NET elevations associated with disease severity [10,36], findings in pediatric patients are more heterogeneous. In neonates, cord blood analyses revealed no increase in NET markers, and similarly, children with acute COVID-19 showed absent or minimal systemic NET release [26,29]. In respiratory infections, adults typically display harmful NET-driven lung injury [10,36], and similarly in children, NETs may exacerbate chronic inflammation, particularly in patients with CF. Taken together, these observations suggest that children exhibit a generally inert baseline systemic NET activity compared with adults, but once induced, NETs can mediate both beneficial antimicrobial effects and potent inflammatory effects and complications.

These insights carry important clinical implications. NET-associated markers and NET-related gene signatures may complement existing biomarkers in the diagnosis of pediatric sepsis, or other infections, though they require validation with studies performed in larger multicenter cohorts. Assessing NETs includes techniques that aim to provide both qualitative visualization and quantitative assessment of NET formation [37,38]. Interpretation of NET-related results is complicated by substantial methodological variability across studies. NETs can be quantified via ELISA detection of MPO–DNA or NE–DNA complexes, immunofluorescence microscopy of NET structures, flow cytometry detection of citrullinated histones and measurement of circulating cell-free DNA [37]. Each approach has different sensitivities, specificity profiles and is susceptible to technical artifacts [39,40]. To improve cross-study comparability, it is essential to standardize assay selection, reporting parameters and analytical criteria; importantly, normalization of NET-associated markers to neutrophil counts or activation status would provide a more meaningful representation of NET production rather than absolute values alone [37,40].

Therapeutic modulation of NETs, such as DNase therapy or targeting degradation pathways [40,41,42,43], has been previously proposed, but pediatric evidence remains preliminary. An important consideration in pediatric infections is that strategies targeting NETs may weaken early antiviral defenses and interfere with the development of adaptive immunity. NETs have been shown to trap and neutralize viral particles in vitro, and inhibition of NETosis in murine models of COVID-19 infection has been associated with higher viral loads. Additionally, a recent study of Bonilha et al. [44] indicates that pharmacological modulation of NETs can affect T-cell activation, antigen presentation, and proliferation. Given that children’s immune systems are still developing, these dual risks must be carefully considered when evaluating NET-targeted therapies in pediatric populations. These findings indicate that while NET markers may be promising diagnostic biomarkers, data are inconsistent. The variability observed across studies also underscores the urgent need for standardization of NET measurement using the proposed techniques [37].

This review has some limitations, due to the small sample size of most included studies, the predominance of cross-sectional design, the heterogeneity of infections and NET detection methods. Longitudinal data on outcomes were sparse, and therefore a meta-analysis was not feasible. Moreover, several mechanistic insights are derived from in vitro models rather than clinical studies.

## 5. Conclusions

This extensive literature review offers evidence that highlights the relevance of NETs in pediatric infections and identifies the gaps for future research. Most included studies had small sample sizes and a predominance of cross-sectional designs, with considerable heterogeneity in NET detection methods. Evidence from longitudinal studies on clinical outcomes was limited and a meta-analysis was infeasible. Despite these challenges, the findings underscore important gaps for future studies, which should aim to standardize NET detection, evaluate their prognostic value in large prospective cohorts, and explore how fundamental differences in adults shape NET responses in children. Interventional trials assessing the safety and efficacy of NET-modulating therapies will be crucial to determine whether manipulating NET biology can improve outcomes in pediatric infections.

## Figures and Tables

**Table 1:** Baseline characteristics of included studies.

Author (Year)	Protocol Number	Country	Study Design	Sample Size (*n*)	Population	Mean/ Median Age	Infection Context	Sample Type	NET Assessment
Appelgren et al., 2020 ([24])	2010/106	Sweden	Observational	111 children (subset)	Children & adults with CNS infections	10y [IQR: 5–15]	CNS infections (LNB, others)	CSF	DNA/MPO, elastase assays
Carmona-Rivera et al., 2022 ([29])	NCT04582903, NCT03394053 and NCT0361080	Italy/Chile/USA	Observational	250	COVID-19, MIS-C	**Chile MIS-C cohort** 6 y [IQR: 3–11] **Italian MIS-C cohort** 5.8y [IQR: 0.3–12] **Italian CLL cohort** 13 y [IQR: 9–15] **US CLL cohort** 17y [IQR: 14–19.5]	COVID-19 and MIS-C	Serum, skin biopsies	NET remnants, degradation assays
Fitzpatrick et al., 2023 ([25])	NR	USA	Observational	52	Preschool children with recurrent wheezing	**No sensitization group** 34.7 months (14.6) **Sensitization group** 34.3 months (12.1)	Viral analog stimulation	Blood neutrophils	Extracellular DNA, MPO release
Hoppenbrouwers et al., 2018 ([15])	2015–49	The Netherlands	Observational	60	PICU patients with meningococcal sepsis	2 years and 10 months [IQR, 21 months to 9 years])	Meningococcal sepsis	Serum	MPO-DNA ELISA, in vitro assays
King et al., 2021 ([28])	AREST CF protocol	Australia	Observational	76	Children with CF or chronic cough	**CF group** 4.1 y [IQR: 1.8–6.0] **Non-CF group 7 y** [IQR: 1.8–6.7]	Airway inflammation	BAL fluid	Confocal microscopy, NE activity
Krivošíková et al., 2023 ([31])	NR	Slovakia	Observational + mouse model	148	Children with febrile UTI	**UTI group** 0.8 y [IQR: 0.3–1.3] **Control group** 2.4 y [IQR: 0.5–5.2]	Urinary tract infection	Serum and urine	ecDNA, ncDNA, mtDNA, MPO, cathelicidin
Lenz et al., 2022 ([16])	NCT02567305	Germany	Observational	31	Preterm neonates with suspected sepsis	**EONS group** 2 days (1) **Control group** 1.5 days (0.71)	Neonatal sepsis	Plasma	cfDNA, DNase I, nucleosomes, NE, H3Cit
Martínez-Alemán et al., 2020 ([30])	NM11015 and MB16-0002	Mexico	Pilot observational	14	Cystic fibrosis patients	11 y	Pseudomonas aeruginosa	In vitro assays	NET morphology, microscopy
Seery et al., 2021 ([26])	1226/20 and 1720/20	Argentina	Observational	243	Children with COVID-19 or MIS-C	Median 9 y	COVID-19	Plasma	cfDNA, citH3 ELISA
Stiel et al., 2020 ([14])	PV5374	Germany	Observational	491	Neonates at birth	**Infection group GA** 40.14 [IQR: 37.71–40.29 weeks] **Control group GA** 39.43 [IQR: 38.86–40.86 weeks]	Neonatal early-onset sepsis	Cord blood	cfDNA, NE, MPO
Thornton et al., 2013 ([27])	1295/EP	Australia	Observational	24	Children with rAOM	17.9 m [IQR: 9.7–36.0]	Recurrent acute otitis media	Middle ear effusion	IF microscopy

Abbreviations: CF: Cystic Fibrosis; CNS: Central Nervous System; CSF: Cerebrospinal Fluid; ecDNA: extracellular DNA; EONS: early onset neonatal sepsis; GA: gestational age; IF: Immunofluorescence; MIS-C: Multisystem Inflammatory Syndrome in Children; MPO: myeloperoxidase; mtDNA: mitochondrial DNA; ncDNA: nuclear DNA; NET: Neutrophil Extracellular Traps; NR: Not Reported; PICU: Pediatric Intensive Care Unit; PMN: Polymorphonuclear; rAOM: recurrent acute otitis media; ROS: Reactive Oxygen Species; SEM: Scanning Electron Microscopy; UTI: Urinary Tract Infection.

**Table 2:** Quality assessment of included studies with the Newcastle–Ottawa Scale.

Author (Year)	Selection						Comparability	Outcome		Total Score
	Representativeness of the Sample		Sample Size	Non-Respondents		Ascertainment of Exposure	Comparability	Assessment of Outcome	Statistical Test	
Appelgren et al., 2020 ([24])	*		*			*	*	*	*	6/10
Carmona-Rivera et al., 2022 ([29])	*		*			**	**	**	*	9/10
Fitzpatrick et al., 2023 ([25])	*		*			*	*	*	*	6/10
King et al., 2021 ([28])	*		*			**	*	**	*	8/10
Krivošíková et al., 2023 ([31])	*		*	*		**	*	**	*	9/10
Lenz et al., 2021 ([16])	*		*			*	*	*	*	6/10
Seery et al., 2021 ([26])	*		*			**	*	*	*	7/10
Thornton et al., 2013 ([27])	*		*			*	*	*	*	6/10
**Author (Year)**	**Case Definition Adequate**	**Representativeness of Cohort**	**Selection of Controls/Non-Exposed**	**Ascertainment of Exposure**	**Control for Main Confounder**	**Control for Additional Confounder**	**Assessment of Outcome**	**Follow-Up Long Enough**	**Adequacy of Follow-Up**	**Total Score**
Hoppenbrouwers et al., 2018 ([15])	*	*	*	*	*		*		*	7/9
Martínez-Alemán et al., 2020 ([30])	*	*	*	*	*		*		*	7/9
Stiel et al., 2020 ([14])	*	*		*	*		*			5/9

## Data Availability

The raw data supporting the conclusions of this article will be made available by the authors on request.

## Abbreviations

The following abbreviations are used in this manuscript:

BAL	Bronchoalveolar Lavage Fluid
CF	Cystic Fibrosis
COVID-19	Coronavirus Disease 2019
CNS	Central Nervous System
CSF	Cerebrospinal Fluid
ecDNA	Extracellular DNA
ELISA	Enzyme-Linked Immunosorbent Assay
EONS	Early Onset Neonatal Sepsis
GA	Gestational Age
IF	Immunofluorescence
MIS-C	Multisystem Inflammatory Syndrome in Children
MPO	Myeloperoxidase
mtDNA	Mitochondrial DNA
ncDNA	Nuclear DNA
NET	Neutrophil Extracellular Traps
NOS	Newcastle–Ottawa Scale
NR	Not Reported
OSF	Open Science Framework
PICU	Pediatric Intensive Care Unit
PMN	Polymorphonuclear
PRISMA	Preferred Reporting Items for Systematic Reviews and Meta-Analyses
rAOM	Recurrent Acute Otitis Media
ROS	Reactive Oxygen Species
SEM	Scanning Electron Microscopy
UTI	Urinary Tract Infection

## Appendix A

**Table A1:** The initial search in MEDLINE/Pubmed and Scopus.

Database	Search Strategy
Pubmed/MEDLINE	(“extracellular traps”[MeSH Terms] OR (“extracellular”[All Fields] AND “traps”[All Fields]) OR “extracellular traps”[All Fields] OR (“neutrophil”[All Fields] AND “extracellular”[All Fields] AND “traps”[All Fields]) OR “neutrophil extracellular traps”[All Fields] OR (“netw spat econ”[Journal] OR “nets”[All Fields])) AND (“child*”[All Fields] OR (“adolescences”[All Fields] OR “adolescency”[All Fields] OR “adolescent”[MeSH Terms] OR “adolescent”[All Fields] OR “adolescence”[All Fields] OR “adolescents”[All Fields] OR “adolescent s”[All Fields]) OR (“infant”[MeSH Terms] OR “infant”[All Fields] OR “infants”[All Fields] OR “infant s”[All Fields]) OR (“infant, newborn”[MeSH Terms] OR (“infant”[All Fields] AND “newborn”[All Fields]) OR “newborn infant”[All Fields] OR “neonatal”[All Fields] OR “neonate”[All Fields] OR “neonates”[All Fields] OR “neonatality”[All Fields] OR “neonatals”[All Fields] OR “neonate s”[All Fields])) AND (“infect”[All Fields] OR “infectability”[All Fields] OR “infectable”[All Fields] OR “infectant”[All Fields] OR “infectants”[All Fields] OR “infected”[All Fields] OR “infecteds”[All Fields] OR “infectibility”[All Fields] OR “infectible”[All Fields] OR “infecting”[All Fields] OR “infection s”[All Fields] OR “infections”[MeSH Terms] OR “infections”[All Fields] OR “infection”[All Fields] OR “infective”[All Fields] OR “infectiveness”[All Fields] OR “infectives”[All Fields] OR “infectivities”[All Fields] OR “infects”[All Fields] OR “pathogenicity”[MeSH Subheading] OR “pathogenicity”[All Fields] OR “infectivity”[All Fields] OR (“communicable diseases”[MeSH Terms] OR (“communicable”[All Fields] AND “diseases”[All Fields]) OR “communicable diseases”[All Fields] OR (“infectious”[All Fields] AND “disease”[All Fields]) OR “infectious disease”[All Fields]))
Scopus	TITLE-ABS-KEY (“neutrophil extracellular traps” OR “NETs” AND “child*” OR “adolescent*” OR “infant” OR “neonate” AND “infection” OR “infectious disease”)
Truncation (*) was applied to key terms to retrieve all lexical variants and ensure comprehensive search coverage.	

**Table A2:** Excluded studies that have not met the inclusion criteria.

Study	Title	Reasons for Exclusion
Feys 2024	Lower respiratory tract single-cell RNA sequencing and neutrophil extracellular trap profiling of COVID-19-associatedpulmonary aspergillosis: a single centre, retrospective, observational study	Adult population
Zhang 2025	Screening and Identification of Neutrophil Extracellular Trap-related Diagnostic Biomarkers for Pediatric Sepsis by Machine Learning	In vitro study
Byrd 2015	NETosis in neonates: evidence of a ROS-independent pathway in response to fungal challenge	In vitro study
Khaertynov 2020	The severity of netosis inpatients with neonatal sepsis	Foreign language
Dan 2019	Significance of neutrophil extracellular trap and its markers in the early diagnosis of community-acquired pneumonia in children	Foreign language
Cortjens 2016	Neutrophil extracellular traps cause airway obstruction during respiratory syncytial virus disease	In vitro study
Mercado-Evans 2025	Tamm-Horsfall protein augments neutrophil NETosis during urinary tract infection	In vitro study
Grudzinska 2019	Neutrophils in community-acquired pneumonia: parallels in dysfunction at the extremes of age	No relevant biomarker measured
Khan 2019	Progression of Cystic Fibrosis Lung Disease from Childhood to Adulthood: Neutrophils, Neutrophil Extracellular Trap (NET) Formation, and NET Degradation	Non original data- Review
Muraro 2018	Respiratory Syncytial Virus induces the classical ROS-dependent NETosis through PAD-4 and necroptosis pathways activation	No relevant biomarker measured
Yoo D 2014	NET formation induced by Pseudomonas aeruginosa cystic fibrosis isolates measured as release of myeloperoxidase-DNA and neutrophil elastase-DNA complexes	Adult population
Wang 2020	Excessive Neutrophils and Neutrophil Extracellular Traps in COVID-19	No relevant biomarker measured
Chen 2018	Neutrophil extracellular traps promote macrophage pyroptosis in sepsis	In vitro study
Arruda 2024	Kinetics of neutrophil extracellular traps and cytokines in oral mucositis and Candida infection	Adult population
Krinsky 2023	NETosis induction reflects COVID-19 severity and long COVID: insights from a 2-center patient cohort study in Israel	Adult population
Funchal 2015	Respiratory syncytial virus fusion protein promotes TLR-4-dependent neutrophil extracellular trap formation by human neutrophils	Adult population

## References

[1] Brinkmann V., Reichard U., Goosmann C., Fauler B., Uhlemann Y., Weiss D.S., Weinrauch Y., Zychlinsky A. (2004). Neutrophil Extracellular Traps Kill Bacteria. Science.

[2] Chen Z., Gao F. (2025). The Dual Role of Macrophage Extracellular Traps in Host Defense and Disease: Mechanisms and Therapeutic Implications. Biomolecules.

[3] Wang H., Kim S.J., Lei Y., Wang S., Wang H., Huang H., Zhang H., Tsung A. (2024). Neutrophil extracellular traps in homeostasis and disease. Signal Transduct. Target. Ther..

[4] Jorch S.K., Kubes P. (2017). An emerging role for neutrophil extracellular traps in noninfectious disease. Nat. Med..

[5] Papayannopoulos V. (2018). Neutrophil extracellular traps in immunity and disease. Nat. Rev. Immunol..

[6] Lee K.H., Kronbichler A., Park D.D.Y., Park Y.M., Moon H., Kim H., Choi J.H., Choi Y., Shim S., Lyu I.S. (2017). Neutrophil extracellular traps (NETs) in autoimmune diseases: A comprehensive review. Autoimmun. Rev..

[7] Wang W., Su J., Yan M., Pan J., Zhang X. (2023). Neutrophil extracellular traps in autoimmune diseases: Analysis of the knowledge map. Front. Immunol..

[8] Li P., Li M., Lindberg M.R., Kennett M.J., Xiong N., Wang Y. (2010). PAD4 is essential for antibacterial innate immunity mediated by neutrophil extracellular traps. J. Exp. Med..

[9] Czaikoski P.G., Mota J.M.S.C., Nascimento D.C., Sônego F., Castanheira F.V.E.S., Melo P.H., Scortegagna G.T., Silva R.L., Barroso-Sousa R., Souto F.O. (2016). Neutrophil extracellular traps induce organ damage during experimental and clinical sepsis. PLoS ONE.

[10] Middleton E.A., He X.Y., Denorme F., Campbell R.A., Ng D., Salvatore S.P., Mostyka M., Baxter-Stoltzfus A., Borczuk A.C., Loda M. (2020). Neutrophil Extracellular Traps Contribute to Immunothrombosis in COVID-19 Acute Respiratory Distress Syndrome. Blood.

[11] Yost C.C., Cody M.J., Harris E.S., Thornton N.L., McInturff A.M., Martinez M.L., Chandler N.B., Rodesch C.K., Albertine K.H., Petti C.A. (2009). Impaired neutrophil extracellular trap (NET) formation: A novel innate immune deficiency of human neonates. Blood.

[12] Lawrence S.M., Corriden R., Nizet V. (2018). The Ontogeny of a Neutrophil: Mechanisms of Granulopoiesis and Homeostasis. Microbiol. Mol. Biol. Rev..

[13] Lawrence S.M., Corriden R., Nizet V. (2017). Age-appropriate functions and dysfunctions of the neonatal neutrophil. Front. Pediatr..

[14] Stiel C.U., Ebenebe C.U., Trochimiuk M., Pagarols Raluy L., Vincent D., Singer D., Reinshagen K., Boettcher M. (2020). Markers of NETosis Do Not Predict Neonatal Early Onset Sepsis: A Pilot Study. Front. Pediatr..

[15] Hoppenbrouwers T., Boeddha N.P., Ekinci E., Emonts M., Hazelzet J.A., Driessen G.J., De Maat M.P. (2018). Neutrophil Extracellular Traps in Children with Meningococcal Sepsis. Pediatr. Crit. Care Med..

[16] Lenz M., Maiberger T., Armbrust L., Kiwit A., Von der Wense A., Reinshagen K., Elrod J., Boettcher M. (2022). CfDNA and DNases: New Biomarkers of Sepsis in Preterm Neonates—A Pilot Study. Cells.

[17] Page M.J., Mckenzie J.E., Bossuyt P.M., Boutron I., Hoffmann C., Mulrow C.D., Shamseer L., Tetzlaff J.M., Akl E.A., Brennan S.E. (2021). The PRISMA 2020 statement: An updated guideline for reporting systematic reviews Systematic reviews and Meta-Analyses. BMJ.

[18] Ouzzani M., Hammady H., Fedorowicz Z., Elmagarmid A. (2016). Rayyan-a web and mobile app for systematic reviews. Syst. Rev..

[19] Wells G.A., Wells G., Shea B., Shea B., O’Connell D., Peterson J., Welch, Losos M., Tugwell P., Ga S.W. (2014). The Newcastle-Ottawa Scale (NOS) for Assessing the Quality of Nonrandomised Studies in Meta-Analyses.. https://api.semanticscholar.org/CorpusID:79550924.

[20] Modesti P.A., Reboldi G., Cappuccio F.P., Agyemang C., Remuzzi G., Rapi S., Perruolo E., Parati G., ESH Working Group on CV Risk in Low Resource Settings (2016). Panethnic differences in blood pressure in Europe: A systematic review and meta-analysis. PLoS ONE.

[21] Herzog R., Álvarez-Pasquin M.J., Díaz C., Del Barrio J.L., Estrada J.M., Gil Á. (2013). Are healthcare workers intentions to vaccinate related to their knowledge, beliefs and attitudes? A systematic review. BMC Public Health.

[22] Luchini C., Stubbs B., Solmi M., Veronese N. (2017). Assessing the quality of studies in meta-analyses: Advantages and limitations of the Newcastle Ottawa Scale. World J. Meta-Anal..

[23] Blanchard L., Ray S., Law C., Vega-Salas M.J., Bidonde J., Bridge G., Egan M., Petticrew M., Rutter H., Knai C. (2024). The effectiveness, cost-effectiveness and policy processes of regulatory, voluntary and partnership policies to improve food environments: An evidence synthesis. Public Health Res..

[24] Appelgren D., Enocsson H., Skogman B.H., Nordberg M., Perander L., Nyman D., Nyberg C., Knopf J., Muñoz L.E., Sjöwall C. (2020). Neutrophil extracellular traps (NETs) in the cerebrospinal fluid samples from children and adults with central nervous system infections. Cells.

[25] Fitzpatrick A.M., Mohammad A.F., Huang M., Stephenson S.T., Patrignani J., Kamaleswaran R., Grunwell J.R. (2023). Functional immunophenotyping of blood neutrophils identifies novel endotypes of viral response in preschool children with recurrent wheezing. J. Allergy Clin. Immunol..

[26] Seery V., Raiden S.C., Algieri S.C., Grisolía N.A., Filippo D., De Carli N., Di Lallaf S., Cairolib H., Chiolog M.J., Meregalli C.N. (2021). Blood neutrophils from children with COVID-19 exhibit both inflammatory and anti-inflammatory markers. EBioMedicine.

[27] Thornton R.B., Wiertsema S.P., Kirkham L.A.S., Rigby P.J., Vijayasekaran S., Coates H.L., Richmond P.C. (2013). Neutrophil Extracellular Traps and Bacterial Biofilms in Middle Ear Effusion of Children with Recurrent Acute Otitis Media—A Potential Treatment Target. PLoS ONE.

[28] King P.T., Dousha L., Clarke N., Schaefer J., Carzino R., Sharma R., Wan K.L., Anantharajah A., O’Sullivan K., Lu Z.X. (2021). Phagocyte extracellular traps in children with neutrophilic airway inflammation. ERJ Open Res..

[29] Carmona-Rivera C., Zhang Y., Dobbs K., Markowitz T.E., Dalgard C.L., Oler A.J., Claybaugh D.R., Draper D., Truong M., Delmonte O.M. (2022). Multicenter analysis of neutrophil extracellular trap dysregulation in adult and pediatric COVID-19. JCI Insight..

[30] Martínez-Alemán S., Bustamante A.E., Jimenez-Valdes R.J., González G.M., Sánchez-González A. (2020). Pseudomonas aeruginosa isolates from cystic fibrosis patients induce neutrophil extracellular traps with different morphologies that could correlate with their disease severity. Int. J. Med. Microbiol..

[31] Krivošíková K., Šupčíková N., Gaál Kovalčíková A., Janko J., Pastorek M., Celec P., Podracká Ľ., Tóthová Ľ. (2023). Neutrophil extracellular traps in urinary tract infection. Front. Pediatr..

[32] Khan M.A., Ali Z.S., Sweezey N., Grasemann H., Palaniyar N. (2019). Progression of cystic fibrosis lung disease from childhood to adulthood: Neutrophils, neutrophil extracellular trap (NET) formation, and NET degradation. Genes.

[33] Mercado-Evans V., Branthoover H., Chew C., Serchejian C., Saltzman A.B., Mejia M.E., Zulk J.J., Cornax I., Nizet V., Patras K.A. (2025). Tamm-Horsfall protein augments neutrophil NETosis during urinary tract infection. JCI Insight.

[34] Byrd A.S., O’brien X.M., Laforce-Nesbitt S.S., Parisi V., Hirakawa M.P., Bliss J.M., Reichner J.S. (2015). NETosis in neonates: Evidence of a ROS-independent pathway in response to fungal challenge. J. Infect. Dis. Adv. Access.

[35] Zhang G., Zhang K. (2025). Screening and Identification of Neutrophil Extracellular Trap-related Diagnostic Biomarkers for Pediatric Sepsis by Machine Learning. Inflammation.

[36] Mikacenic C., Moore R., Dmyterko V., West T.E., Altemeier W.A., Liles W.C., Lood C. (2018). Neutrophil extracellular traps (NETs) are increased in the alveolar spaces of patients with ventilator-associated pneumonia. Crit. Care.

[37] Stoimenou M., Tzoros G., Skendros P., Chrysanthopoulou A. (2022). Methods for the Assessment of NET Formation: From Neutrophil Biology to Translational Research. Int. J. Mol. Sci..

[38] Lee K.H., Cavanaugh L., Leung H., Yan F., Ahmadi Z., Chong B.H., Passam F. (2018). Quantification of NETs-associated markers by flow cytometry and serum assays in patients with thrombosis and sepsis. Int. J. Lab. Hematol..

[39] Henneck T., Krüger C., Nerlich A., Langer M., Fingerhut L., Bonilla M.C., Meurer M., von den Berg S., de Buhr N., Branitzki-Heinemann K. (2023). Comparison of NET quantification methods based on immunofluorescence microscopy: Hand-counting, semi-automated and automated evaluations. Heliyon.

[40] Retter A., Singer M., Annane D. (2025). “The NET effect”: Neutrophil extracellular traps—A potential key component of the dysregulated host immune response in sepsis. Crit. Care.

[41] Espiritu A., O’Sullivan K.M. (2025). A Web of Challenges: The Therapeutic Struggle to Target NETs in Disease. Int. J. Mol. Sci..

[42] Tonello S., Vercellino N., D’Onghia D., Fracchia A., Caria G., Sola D., Tillio P.A., Sainaghi P.P., Colangelo D. (2025). Extracellular Traps in Inflammation: Pathways and Therapeutic Targets. Life.

[43] Mutua V., Gershwin L.J. (2021). A Review of Neutrophil Extracellular Traps (NETs) in Disease: Potential Anti-NETs Therapeutics. Clin. Rev. Allergy Immunol..

[44] Bonilha C.S., Veras F.P., dos Santos Ramos A., Gomes G.F., Rodrigues Lemes R.M., Arruda E., Alves-Filho J.C., Cunha T.M., Cunha F.Q. (2025). PAD4 inhibition impacts immune responses in SARS-CoV-2 infection. Mucosal Immunol..

